# The Learning Styles Myth is Thriving in Higher Education

**DOI:** 10.3389/fpsyg.2015.01908

**Published:** 2015-12-15

**Authors:** Philip M. Newton

**Affiliations:** Swansea University Medical SchoolSwansea, UK

**Keywords:** learning styles, evidence-based education, Vark, Kolb, higher education

## Abstract

The existence of ‘Learning Styles’ is a common ‘neuromyth’, and their use in all forms of education has been thoroughly and repeatedly discredited in the research literature. However, anecdotal evidence suggests that their use remains widespread. This perspective article is an attempt to understand if and why the myth of Learning Styles persists. I have done this by analyzing the current research literature to capture the picture that an educator would encounter were they to search for “Learning Styles” with the intent of determining whether the research evidence supported their use. The overwhelming majority (89%) of recent research papers, listed in the ERIC and PubMed research databases, implicitly or directly endorse the use of Learning Styles in Higher Education. These papers are dominated by the VAK and Kolb Learning Styles inventories. These presence of these papers in the pedagogical literature demonstrates that an educator, attempting to take an evidence-based approach to education, would be presented with a strong yet misleading message that the use of Learning Styles is endorsed by the current research literature. This has potentially negative consequences for students and for the field of education research.

## Introduction

Davies (1999) called for an ‘evidence-based’ approach to education, arguing that the design of new interventions in education was not sufficiently informed by rigorous evidence and that new interventions are then poorly evaluated. Although this was one in a series of such calls, the very idea of evidence-based education continues to be criticized (e.g., see Biesta, 2010), often on the basis that education is complex (allegedly more so than medicine, to which it is often compared). For example, what does it mean to say that something has ‘worked’, and for whom has it ‘worked’ and in what context?

Despite this complexity, there are some concepts in education for which there is abundant clear evidence to show that they are *not* effective. One of these is Learning Styles, such as the ‘VAK’ classification, which classifies individuals as one or more of ‘Visual, Auditory, or Kinesthetic’ learners (Geake, 2008). Other classifications include those by (separately) Kolb, Felder and Honey and Mumford; in total there are over 70 different classification systems (Coffield et al., 2004). The concept of ‘Learning Styles’ as an educational tool is fairly straightforward, and follows three steps: (1) individuals will express a preference regarding their ‘style’ of learning, (2) individuals show differences in their ability to learn about certain types of information (e.g., some may be better at learning to discriminate between sounds while others may be better at discriminating between pictures), and (3) the ‘matching’ of instructional design to an individual’s Learning Style, as designated by one of the aforementioned classifications, will result in better educational outcomes (e.g., visual learners should have information presented visually, while auditory learners would do better with an emphasis on audio).

The utility of step 2 for education is debatable, as most learning is constructed from multiple types of information, and to acquire ‘meaning’ and ‘understanding’ arguably goes beyond a specific sensory domain. However, it is the last step, the ‘matching hypothesis’, where the concept of Learning Styles completely falls down. A comprehensive review by Pashler et al. (2008) determined that there was no evidence to support the use of Learning Styles in education, based upon a lack of evidence to support ‘matching’. Coffield et al. (2004) reviewed the literature pertaining to 71 different Learning Styles classifications, with the aim of answering the question “should we be using them in post-16 education.” The answer was a resounding ‘no’.

The use of an ineffective educational technique is potentially associated with harm – students who are labeled as having a dominant Learning Style (e.g., ‘visual learners’) may then choose not to pursue subjects which they perceive as being dominated by a different learning style (e.g., music), or may develop a false sense of confidence in their abilities to master subjects which they perceive as matching their style. Perhaps most importantly, the use of ineffective techniques such as Learning Styles can detract from the use of techniques which *are* demonstrably effective (Riener and Willingham, 2010; Willingham et al., 2015).

Despite this, amongst educators, there appears to be widespread belief in the use of Learning Styles. A survey by Dekker et al. (2012) showed that 93% of UK schoolteachers believed the (unsupported) statement that “individuals learn better when they receive information in their preferred Learning Style”. Follow-up studies have shown similar results in other countries (Howard-Jones, 2014). A study conducted using faculty in Higher Education in the USA found similar results, with 64% rating ‘yes’ to the statement “does teaching to a student’s learning style enhance learning” (Dandy and Bendersky, 2014). This is reflected at the institutional level – a survey of 39 Higher Education institutions in the US found that 29 of them (72%) taught ‘learning style theory’ as part of faculty development for online teachers (Meyer and Murrell, 2014).

Learning Styles have been designated a ‘neuromyth’ (Lilienfeld et al., 2011, p. 92; Dekker et al., 2012; Howard-Jones, 2014) and the lack of evidence to support them has been the subject of reviews and commentaries (Riener and Willingham, 2010; Rohrer and Pashler, 2012; Willingham et al., 2015). Alongside this formal literature are blogs and online videos debunking the ‘myth.’ I wrote one myself, motivated, as I am sure others have been, by my personal experience of meeting numerous students and educators who accepted the concept of Learning Styles as an established, textbook principle. However, with the wealth of strong research studies and social media, it seemed reasonable to hypothesize that the use of Learning Styles may now be in decline, and that this would be seen most keenly in the current research literature.

Alternately, Learning Styles may represent the educational equivalent of homeopathy: a medical concept for which no evidence exists, yet in which belief and use persists. There has been a significant body of research aimed at understanding why such beliefs persist, a simple summary of which is that people often seek out information which aligns with their existing worldview, akin to a prospective confirmation bias (Colombo et al., 2015). Confirmation bias has been suggested as one reason why Learning Styles and other myths appear to persist (Riener and Willingham, 2010; Pasquinelli, 2012).

Intuitively, there is much that is attractive about the concept of Learning Styles. People are obviously different and Learning Styles appear to offer educators a way to accommodate individual learner differences. They also allow individuals to self-test and determine what ‘type’ of learner they are. These intuitive attractions may ‘set up’ an educator to fall into the trap of confirmation bias – approaching the research literature having already formed a view that Learning Styles are ‘a good thing’. Therefore, I also set out to characterize the picture an educator would encounter were they to search the education research literature for evidence to support, or not, the use of Learning Styles.

## Methodology

Two major databases of life sciences/education research were used as the datasets. PubMed is a database of research publications in the life sciences and biomedicine^1^ While ERIC (Education Information Resources Center) is ‘an online library of education research and information’^2^

A search of the PubMed database^3^ was carried out for the term “learning styles”, with the date range July 23, 2013 to July 23, 2015 (to reflect current research). Only papers studying Higher Education were selected for analysis. The term “learning styles” was also used to search the ERIC database, with results filtered to be positive for the criteria ‘peer reviewed’ and ‘Higher Education’ for 2015, then 2014, then 2013 (July–December).

The analysis was restricted to Higher Education on the basis that (1) one of the most comprehensive reviews regarding the use of Learning Styles in education was focused specifically on post-16 education (Coffield et al., 2004) and (2) a lecturer in Higher Education is normally appointed as a subject-matter expert on the basis of their research expertise, and so would normally be familiar with using research literature.

For every search result, the following questions were asked (further detail below) –

• (Inclusion criteria for further analysis)
  ◦ Was the study *about* Learning Styles?
  ◦ Were participants students/staff in Higher Education or beyond?
  ◦ Was the full text in English?
• What was the specific study population (e.g., medical students)?
• In which country was the study conducted?
• What Learning Style(s) was being used or tested?
• Does the study begin with positive view toward Learning Styles?
• Does the study conclude with positive view toward Learning Styles?
• Does the study test the matching hypothesis as put forward by Pashler et al. (2008)?
• Do the study results challenge the conclusions of Pashler/Coffield?

This study was aimed at providing a snapshot of the Learning Styles research available to the ‘casual’ inquirer – an academic considering the use of these methods in their teaching. Thus the questions asked were initially of the study abstract. If the answers were clear from the abstract, then the full text was not consulted. If the answers were not clear from the abstract, then the full text was consulted. Only full text papers that were freely available were consulted; if a subscription or payment was required, then the result was not included because access to them would vary considerably between individual educators.

Details about the questions asked:

### Was the Study About the Use of Learning Styles?

The term ‘Learning Styles’ was taken to mean of one of the 71 Learning Styles inventories described in Figure 4 of Coffield et al. (2004). The studies analyzed had to use Learning Styles in a manner which attempted to understand something about student learning (including the training of educators).

Examples of studies which did not meet this criterion included those about ‘learning styles’ rather than ‘Learning Styles’, i.e., they used the term learning styles as a normal grammatical construct or talked in broad terms about ‘learning styles’. For example, an article may discuss the need to ‘accommodate different learning styles’ without it being conclusively clear that this referred to Learning Styles as defined by Coffield et al. (2004).

### What Learning Style is Being Used or Tested?

This was classified using the aforementioned review by Coffield et al. (2004), with appropriate adaption [e.g., studies using variations on the VAK Learning Styles classification (such as ‘VARK’ and ‘VAKT’) were all grouped together].

### Does the Study Begin with Positive View Toward Learning Styles?

**Yes -** Did the study start with a premise that the use/identification of Learning Styles was a useful aim. This could be implicit (e.g., the premise of the study includes an assumption that it is useful to identify a learner’s Learning Style even if the finding is then that Learning Styles are not effective in that context).

**No –** The study was setting out to test Learning Styles themselves, e.g., to determine whether their use was valid.

### Does the Study Conclude with Positive View Toward Learning Styles?

**Yes –** The study concluded that the use of Learning Styles was effective for student learning. This again could be implicit – for example, studies where a group of participants are classified using a Learning Styles inventory and the conclusion is then that the dominant Learning Styles in this group are X and Y.

**No –** The study concluded that the use of Learning Styles by educators was not effective for student learning.

### Test Matching?

Did the study test the ‘matching hypothesis’ as described in Pashler et al. (2008). That is, does matching instruction to a students Learning Style result in improved outcomes?

### Contradict Pashler/Coffield

Do the research findings challenge the conclusions drawn by Pashler et al. (2008) and Coffield et al. (2004)? That is, does the reported evidence support the idea that matching instructional design to individual student Learning Style is effective?

## Results

### Number of Search Results

**FIGURE 1:**
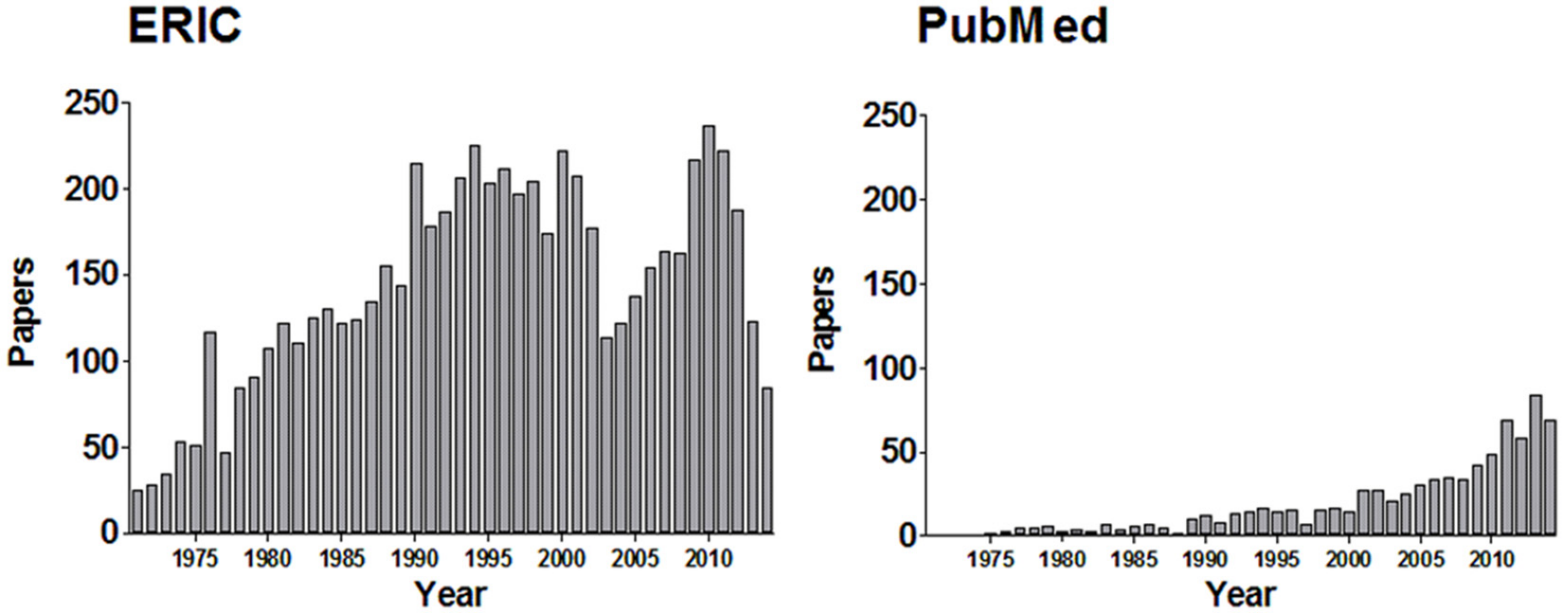
**Research database search results for the term “Learning Styles” filtered by individual calendar year**.

The data for search results are shown in **Figure 1.** The ERIC research database returned more results than PubMed, but both demonstrate that an educator conducting a simple search for “learning styles” would be presented with abundant, modern, results, although there is a suggestion that the numbers of studies may be declining in the ERIC database. These data do not necessarily reflect an increase in use of Learning Styles or in Learning Styles research; there may be a concurrent increase in the total number of publications listed in these databases.

### Endorsement of Learning Styles

For ERIC, the initial search for recent papers returned 110 unique results, of which 54 met the inclusion criteria. For PubMed, the initial search returned 126 unique results; 57 of these met the inclusion criteria. ‘Unique results’ refers to results that appeared only once within that database. Two studies were present in both databases and so were excluded from any pooled analysis (*N* = 109). The results of the subsequent analysis are shown in **Table 1.**

**Table 1:** The majority of current research findings in the PubMed and ERIC databases endorse the use of Learning Styles.

Analysis	All (109)	ERIC (54)	PubMed (57)
Positive intent	103 (94%)	51 (94%)	54 (95%)
Positive outcome	97 (89%)	49 (91%)	50 (85%)
Test matching	1	1	0
Contradict Pashler/Coffield	0	0	0


*Only one study tested the ‘matching hypothesis’ and found that matching instructional type to individual students Learning Style had no effect, as has been repeatedly demonstrated previously (Pashler et al., 2008).*

Most (94%) of the current research papers start out with a positive view of Learning Styles, despite the aforementioned research which discredits their use. Six papers started out with positive intent, but reached a negative conclusion regarding the use of Learning Styles.

One study, not shown in **Table 1**, described testing a ‘matching hypothesis’ and appeared to show that matching had some benefit, but from the data presented it was not clear whether it was truly a matching hypothesis as proposed by Pashler et al. (2008), or which specific Learning Style inventory was being tested, or how the data were analyzed (Surjono, 2015).

### Type of Learning Style

The majority of papers featured a single Learning Style. The Kolb classification accounted for 34% of all papers, while VAK-type classifications accounted for 33%. The Felder–Silverman (and Felder–Soloman) classifications accounted for (12%). Other featured classifications included Vermunt (2 of the 109 studies) Honey and Mumford (itself derived from Kolb) (3), Grasha–Reischmann (4), Myers–Briggs type (4), Biggs Study Process Questionnaire (2), Dunn and Dunn (1), and Gregorc (1). Five publications addressed Learning Styles generally.

### Type of Participant

The studies included participants from across a wide variety of disciplines. Notably, for the studies obtained using PubMed, students in health professions programs (medicine, nursing, dentistry, etc.,) dominated, accounting for a total of 53 out of 57 studies (93%).

### Country of Origin

The studies represented a total of 29 different countries around the world, with the single biggest contribution coming from the USA (41 studies, 33%) followed by Turkey (10, 8%).

### Excluded Studies

Approximately half (125) of the search results were not analyzed. More than half of these (66) were not demonstrably about ‘Learning Styles’, while the full text was not available for 22. Other reasons for exclusion included study populations other than students/teachers in higher education (12), non-English language (6) and the use of ‘Learning Styles’ other than those listed in Coffield et al. (2004) (5).

## Discussion

The data presented here demonstrate that the use of Learning Styles is thriving in Higher Education. This result is somewhat surprising given the rigorous research (Coffield et al., 2004; Pashler et al., 2008) demonstrating the ineffectiveness of Learning Styles, alongside an abundance of critical material in social media. The use of Learning Styles may cause harm through ‘pigeon-holing’ and the diversion of resources away from evidence-based practices (Riener and Willingham, 2010; Willingham et al., 2015). Why, then, is the recent research literature so overwhelmingly misleading?

The literature on cognitive bias indicates that we will seek, or at least be sympathetic to, information which confirms our existing worldview. Confirmation bias has been suggested as a reason for the success of Learning Styles (Riener and Willingham, 2010; Pasquinelli, 2012) and there is much that is attractive about the basic idea of Learning Styles. Thus an educator might reasonably approach the literature with an expectation that Learning Styles are a useful tool. The present study demonstrates that this view would be overwhelmingly confirmed, thus encouraging and perpetuating the use of Learning Styles.

There is another interpretation – if the majority of studies endorse the use of Learning Styles, then maybe Coffield et al. (2004) and Pashler et al. (2008) are wrong? The lack of any evidence base to support the use of Learning Styles was acknowledged by some of the studies found here, despite their overall endorsement of the use of Learning Styles. Some even cite the works of Coffield et al. (2004), Pashler et al. (2008), Willingham et al. (2015), and others. Some engage the literature and defend the use of Learning Styles as identifying ‘learner preferences’ rather than a basis for matching instruction, or as a prompt for students to reflect on how they learn. Overall though, most studies appear generally uncritical – a very common approach is to simply apply a Learning Style classification to a particular type of student, and then make recommendations based upon the findings. These studies do not really engage the aforementioned evidence.

There is an obvious limitation to the findings presented here – a single researcher has analyzed the papers and made a subjective judgment as to whether or not they endorse the use of Learning Styles. This methodology may lack sufficient rigor to be fully endorsed by many advocates of evidence-based education. However, the papers are all in the **Supplementary Data Sheet S1**. I approached the analysis with an open mind, half expecting to find an abundance of studies decrying Learning Styles. In the end this was overwhelmingly not the case, and I found it relatively straightforward to decide whether a study endorsed the use of Learning Styles.

## Conclusion And Recommendations

Learning Styles do not work, yet the current research literature is full of papers which advocate their use. This undermines education as a research field and likely has a negative impact on students.

If you have got this far in reading this perspective, you likely care about education, and about education research. It is in everyone’s interests for educational research and resources – time, money, effort, to be directed toward those educational interventions which demonstrably improve student learning, and away from those which do not. Take a second to run a Google search on your own institution – put in the domain name – youruniversity.edu or.ac.uk or whatever it is, alongside the term “learning styles”. Chances are, something will come up. Start there!

## Conflict of Interest Statement

The author declares that the research was conducted in the absence of any commercial or financial relationships that could be construed as a potential conflict of interest.

## Footnotes

1 www.pubmed.com.

2 www.eric.ed.gov.

3 http://www.ncbi.nlm.nih.gov/pubmed
